# A New Medical Organisation in Erie County

**Published:** 1900-06

**Authors:** 


A Monthly Review of Medicine and Surgery.

*EDITOR:*

WILLIAM WARREN POTTER, M. D.

All communications, whether of a literary or business nature, books for review and exchanges should be addressed to the editor: 284 FRANKLIN STREET, BUFFALO, N. Y.

VOL. XXXIX.—LV. JUNE, 1900. NO. 11.

### A NEW MEDICAL ORGANISATION IN ERIE COUNTY.

THE incorporation of the New York State Medical Association by the legislature last winter, has had the effect of bringing the independent county organisations existing at the time under the power and dictation of the state association. Furthermore, the state association has sent out orders for the organisation of county associations where such have not existed heretofore. The creation of an Erie County Medical Association was demanded by the powers that be, and a meeting was held in the Y. M. C. A. hall for the purpose, May 21, 1900. Thus, another medical society is planned for this part of the state, already prolific and fertile in this respect.

The object of this new organisation, according to the statement of friends of the movement, is primarily to enable physicians to join the American Medical Association. This would seem to be an insufficient reason, as scores of practitioners have been received with open arms by the parent association since the state medical society took its advanced and manly stand on the code question in 1884. Some other motive is apparently behind this move, which most probably is to bolster up the state association in its warfare on the rights and privileges granted the Medical Society of the State of New York by past legislatures. The passage of the state examining board law in 1890 put the state on record as recognising all physicians graduating from a regularly incorporated medical college. When, therefore, this law was put in force, it changed the whole aspect of the code question in this state at least, and physicians qualified to practise medicine were equal before the law.

Physicians not identified with the state association or the state medical society have been able to join the American Medical Association, and hence this excuse for organising separate county associations must fall. As far as the scientific work of county societies is concerned, the Medical Society of the County of Erie, likewise the county societies of Western New York, notably those of Chautauqua and Cattaragus, have done excellent service and deserve the good will and patronage of all physicians irrespective of code or affiliation.

Besides the local city and county medical societies, public and private, Erie County is deeply interested in the Central New York Medical Association and the fourth district branch of the State Association. The attendance upon these societies already existing is not what it should be, and they lack in nearly all of them that spontaneity of interest and attention which go so far toward making a successful medical meeting. To further divide the small coterie of workers who have been active in the societies already existing, would be unwise and in our opinion prejudicial to the best interests of the profession. Would it not be better to concentrate all the strength and enthusiasm on the older societies and show that the profession can be united and strong in its organisation? If no need has existed for a county association to this late day, it is safe to infer that no pressing need is at hand just now.

